# Mutation specific antibodies: tool or dinosaur?

**DOI:** 10.18632/oncotarget.633

**Published:** 2012-08-30

**Authors:** Andreas von Deimling, Felix Sahm, David Capper

**Affiliations:** Department of Neuropathology, Institute of Pathology, Ruprecht-Karls-Universität Heidelberg, and Clinical Cooperation Unit Neuropathology, German Cancer Research Center (DKFZ), Germany; Department of Neuropathology, Institute of Pathology, Ruprecht-Karls-Universität Heidelberg, and Clinical Cooperation Unit Neuropathology, German Cancer Research Center (DKFZ), Germany; Department of Neuropathology, Institute of Pathology, Ruprecht-Karls-Universität Heidelberg, and Clinical Cooperation Unit Neuropathology, German Cancer Research Center (DKFZ), Germany

Cancer patients will receive targeting therapy based on knowledge of deregulated genes or signaling pathways. Treatment will be individually designed on genome, transcriptome and proteome data. What a promise! Pathologists will sight with relief as this rids them of the pressing task to identify targets for therapies. Is there still need for old fashioned immunohistochemistry with machinery around providing answers to all questions whether asked or not?

One of the current hot targets is the BRAF gene. By far the most numerous BRAF alteration is a single point mutation resulting in an amino acid exchange in position 600 dubbed V600E. The substance PLX4032/RG7204 [1] has shown promise in the treatment of malignant melanoma with mutations in BRAF around amino acid position 600 and the drug termed Vemurafenib/Zelboraf consequently received FDA approval for this application. BRAF mutations are not restricted to melanoma [2]. V600E frequently occurs in thyroid-, ovarian-, colon-carcinomas, hairy cell leukemia, Langerhans cell histiocytosis, Erdheim Chester disease, pleomorphic xanthoastrocytoma and ganglioglioma. Many more tumor entities carry V600E with an incidence below 5%. All these patients are potential candidates for therapy with Vemurafenib/Zelboraf or related drugs. Thus, the pressure for identification of the target in the population of potentially eligible patents is mounting. Fortunately, the assessment of the relevant region of BRAF requires DNA analysis only of a single PCR product. This currently is done routinely in many clinical centers worldwide for patients with melanoma and other tumor entities with very high V600E incidences. However, what are we doing with patients having a probability of maybe 4% or less to carry V600E? Will these patients be identified and drugged accordingly or included properly in trials? The likely answer is “No”! It is doubtful that the majority of cancer patients currently will be assessed for this mutation due to financial and logistic restrictions. Similar restrictions also darken the vision of all cancer patients receiving exome/genome sequencing followed by targeted therapy for most drugs still to be developed.

Recently, a monoclonal antibody specifically recognizing the V600E mutation has been developed [3] promising simple rapid and inexpensive detection of V600E by routine immunohistochemistry widely available in diagnostic routine pathology institutions. The obvious question arises how this novel tool compares to the current gold standard DNA analysis. Not unexpectedly, there are two answers to this. For the detection of the V600E mutation only, a series of studies on different tumor entities has shown that the antibody is highly sensitive and specific [4-6]. In fact, rare divergent results between BRAF DNA sequencing and V600E immunohistochemistry usually resolve in favor of the antibody based results. This is due to the ability of the antibody to clearly detect small cell groups in otherwise healthy tissue evading recognition by sequencing due to insufficient copy number of mutant alleles. On the other hand, the antibody does not detect the V600K mutation which is second in frequency to V600E and patients with that mutation likely also respond to Vemurafenib. Thus, the pros and cons in respect to detection of the relevant mutations with antibodies vs. sequencing appear more or less balanced. Currently, a strategy involving immunohistochemistry based screening for V600E mutation supplemented by DNA analysis in those entities at danger of substantial numbers of patients with V600K mutations being missed might be a good choice. This would ensure identification of the majority of therapy relevant mutations in tumors with low BRAF mutation frequencies, and detection of all therapy susceptible BRAF mutations in tumors with high BRAF mutation frequencies.

Moreover, the antibodies ability to identify single cells with the V600E mutation and to provide information on the quantity of mutant protein opens a range of novel opportunities beyond qualitative diagnostic application. Single cell identification allows addressing questions regarding the origin and evolution of tumors. This is of special interest in inhomogeneous tissues such as Langerhans cell histiocytosis with the antibody allowing identifying the neoplastic element [7, 8]. Vice versa, based on sequencing V600E heterogeneity has been observed within individual solid tumors, a finding which V600E immunohistochemistry does not appear to substantiate. Early and very small tumorous lesions in human tissues or animal models can be analyzed and tightly monitored. It will be very interesting to see, whether the amount of translated mutant protein likely to correlate with staining intensity has influence on therapy response. Such constellation is well established for other antigens such as HER2/neu expression in breast cancer and corresponding targeting therapy i.e. Herceptin.

Will there be many more mutation specific antibodies in the future? This very much depends on the recognition of genes exhibiting highly recurrent mutations. In terms of frequency BRAFV600E is the current leader of the pack. IDH1R132H being the dominant mutation in diffuse gliomas and also frequent in AML, chondrosarcomas and cholangiocarcinomas already can be identified by mutation specific antibodies [9]. A very good candidate is JAK2V617F present in myeloproliferative disease. Of course, antibodies recognizing BRAFV600K and IDH1R132C, both second in frequency to their dominant counterparts, would nicely complement the diagnostic armory. Mutation specific antibodies demonstrate the benefit of merging molecular findings with traditional diagnostic expertise. Whether this approach is of sustainable benefit has to be seen. Convincing data on colon carcinoma will be demonstrated at ESMO 2012 with the conclusion to screen patients for BRAFV600E for enrolment in an upcoming clinical trial combining BRAF and EGFR inhibitors [10].
